# Determinants of Arsenic Metabolism: Blood Arsenic Metabolites, Plasma Folate, Cobalamin, and Homocysteine Concentrations in Maternal–Newborn Pairs

**DOI:** 10.1289/ehp.9906

**Published:** 2007-06-28

**Authors:** Marni Hall, Mary Gamble, Vesna Slavkovich, Xinhua Liu, Diane Levy, Zhongqi Cheng, Alexander van Geen, Mahammad Yunus, Mahfuzar Rahman, J. Richard Pilsner, Joseph Graziano

**Affiliations:** 1 Department of Environmental Health Sciences, Columbia University Medical Center, New York, New York, USA; 2 Department of Biostatistics, Mailman School of Public Health, Columbia University Medical Center, New York, New York, USA; 3 Lamont-Doherty Earth Observatory, Columbia University, New York, New York, USA; 4 ICDDR, B: Centre for Health and Population Research, Dhaka, Bangladesh; 5 Department of Pharmacology, College of Physicians and Surgeons, Columbia University, New York, New York, USA

**Keywords:** arsenic, B_12_, blood arsenic metabolites, DMA, folate, homocysteine, maternal, MMA, newborn

## Abstract

**Background:**

In Bangladesh, tens of millions of people have been consuming waterborne arsenic for decades. The extent to which As is transported to the fetus during pregnancy has not been well characterized.

**Objectives:**

We therefore conducted a study of 101 pregnant women who gave birth in Matlab, Bangladesh.

**Methods:**

Maternal and cord blood pairs were collected and concentrations of total As were analyzed for 101 pairs, and As metabolites for 30 pairs. Maternal urinary As metabolites and plasma folate, cobalamin, and homocysteine levels in maternal cord pairs were also measured. Household tube well–water As concentrations exceeded the World Health Organization guideline of 10 μg/L in 38% of the cases.

**Results:**

We observed strong associations between maternal and cord blood concentrations of total As (*r* = 0.93, *p* < 0.0001). Maternal and cord blood arsenic metabolites (*n* = 30) were also strongly correlated: in dimethylarsinate (DMA) (*r* = 0.94, *p* < 0.0001), monomethylarsonate (*r* = 0.80, *p* < 0.0001), arsenite (As^+3^) (*r* = 0.80, *p* < 0.0001), and arsenate (As^+5^) (*r* = 0.89, *p* < 0.0001). Maternal homocysteine was a strong predictor of %DMA in maternal urine, maternal blood, and cord blood (β = −6.2, *p* < 0.02; β = −10.9, *p* < 0.04; and β = −13.7, *p* < 0.04, respectively). Maternal folate was inversely associated with maternal blood As^5+^ (β = 0.56, *p* < 0.05), and maternal cobalamin was inversely associated with cord blood As^5+^ (β = −1.2, *p* < 0.01).

**Conclusions:**

We conclude that exposure to all metabolites of inorganic As occurs in the prenatal period.

Exposure to arsenic-contaminated drinking water represents one of the largest public health poisonings in history, affecting tens of millions of people worldwide (Chowdhury 2004). Bangladesh is one of the most severely affected regions. Adverse health effects associated with chronic exposure to As-contaminated water include skin, bladder, kidney, and liver cancers, diabetes, and hypertension [National Research Council (NRC) 1999]. Reports in the literature suggest that inorganic As (InAs) also poses increased reproductive and developmental risks in animals, though few human studies have focused on these outcomes (DeSesso et al. 1998; Waalkes et al. 2004). Relatively little is known about transplacental transfer of As. Several studies have addressed the impact of human prenatal As exposure, and have reported increases in miscarriage, stillbirths, and congenital heart disease (Ahmad et al. 2001; Borzsonyi et al. 1992; Hopenhayn-Rich et al. 2000).

The As in drinking water is typically inorganic, and can be present either as As^+3^ (arsenite) or As^+5^ (arsenate). In Bangladesh, As is primarily in the As^+3^ form, though there are reports of a significant +5 fraction in other parts of the Bengal Delta (Zheng et al. 2004). The metabolism of As in humans involves a series of reactions that alternates reduction of pentavalent As species to the trivalent state with oxidative methylation steps (Vahter and Concha 2001). In humans this process results in distinct As species, including As^+3^, As^+5^, monomethylarsonous acid (MMA^+3^), monomethylarsonic acid (MMA^+5^) and dimethlyarsinic acid (DMA^+5^), each with unique toxicology (Aposhian et al. 2004). The trivalent As forms, which have a higher affinity for thiol groups (Vahter and Concha 2001), are more cytoxic and genotoxic than pentavalent forms. Individuals who accumulate the trivalent intermediates are thought to be at greater risk of As-induced diseases (Rossman et al. 2004; Thomas et al. 2004). A series of studies from Taiwan indicate that individuals who excrete relatively lower proportions of urinary DMA^(+3) + (+5)^ are at increased risk for skin and bladder cancers and for peripheral vascular disease (Chen et al. 2003a, 2003b; Hsueh et al. 1997; Tseng et al. 2005; Yu et al. 2000).

Interindividual differences in metabolism of As are thought to account for some of the observed variation in susceptibility to As-related diseases (NRC 1999; Vahter 2000). Low body mass index (Milton et al. 2004) and reduced methylation capacity (Davis and Uthus 2004; Gamble et al. 2005b) are thought to impair As metabolism and enhance toxicity. Arsenic is methylated by *S*-adenosylmethionine (SAM) via one-carbon metabolism, a biochemical pathway that uses folate for recruitment of methyl groups and cobalamin (B_12_) as a cofactor. We previously reported a high prevalence of hyperhomocysteinemia in Bangladesh that was found to be associated with reduced As methylation (Gamble et al. 2005a, 2005b). We also found that As methylation can be increased with folic acid supplementation (Gamble et al. 2006.)

Early manifestations of arsenicosis, such as skin lesions, occur in adults after many years of exposure, whereas As-induced cancers and cardiovascular disease take decades to develop. Little is known about the comparable latency periods in children. Anecdotally, we have observed As-induced skin lesions in preschool-age children in Bangladesh, and have become concerned for children who are now being born to a generation of mothers who have been exposed to As for much or all of their lives. In addition, we recently reported that young children exposed to As in drinking water exhibit dose-dependent intellectual impairment (Wasserman et al. 2004). These observations strongly suggest that As exposure *in utero* and/or during early childhood may be contributing to As-induced health effects in children. To determine the early health effects associated with As, prenatal exposures must be better characterized. The purpose of this study was to characterize the As metabolic profiles in maternal–cord blood pairs, and to determine the extent to which maternal folate and B_12_ status influences maternal As metabolism. Here we report total blood As, urinary As metabolites, folate, B_12_, and homocysteine findings in 101 maternal–cord blood pairs derived from births in Matlab, Bangladesh, an area with a high prevalence of elevated As concentrations in tube-well water. We also describe blood As metabolites in the 30 maternal–cord pairs that had the highest total blood As.

## Methods

### Study participants

This study was approved by the Institutional Review Board of the Columbia University Medical Center and the Ethical Review Committee of the International Centre for Diarrheal Disease Research-Bangladesh (ICDDR-B). Between December 2004 and April 2005, pregnant women who appeared at the main hospital in Matlab, Bangladesh, were asked to participate in the study by the attending physican. For the past 30 years, ICDDR-B has carried out a disease surveillance program in Matlab, an area that is roughly 20 km north of Araihazar. Matlab is a region that has variable concentrations of As in groundwater, and where relatively little As remediation had been carried out at the time of the study. Through the efforts of ICDDR-B, roughly half of all women in Matlab now deliver their babies in the main hospital or in one of several medical field stations in the region.

A convenience sample of 104 pregnant women participated in the study, all of whom signed written informed consent. This group is not necessarily representative of all women in the study region. The project benefited from the ICDDR-B health and demographic surveillance system (HDSS) in Matlab. HDSS records all vital events, such as births, deaths, marriages, including the date of the last menstrual period, pregnancies, and pregnancy outcomes, as well as in- and out-migration. During their pregnancies, all women were provided with daily iron (60 mg/day) and folic acid (300 μg/day) supplements.

### Sample collection and storage

Near the time of delivery, women were asked to provide written informed consent, to answer a brief questionnaire, to provide a blood and urine sample before delivery, and to allow a blood sample to be obtained from the discarded umbilical cord after the birth of the baby. The maternal and cord blood samples were typically taken within 1–2 hr of each other. The questionnaire was administered by a trained nurse, who also obtained the blood and urine samples. The length and weight of the baby at birth were also recorded. In three cases, cord blood was not collected; we therefore report on a sample size of 101.

Blood samples were processed and frozen at −80°C immediately in the hospital laboratory. Samples were collected in EDTA vacutainers; serum was immediately separated for folate, B_12_, and homocysteine analyses. Urine samples were also collected in acid-washed tubes and frozen at −80°C immediately. All samples were ultimately shipped on dry ice to the Mailman School of Public Health at Columbia University, and analyzed in the Trace Metals Core Laboratory; shipment required approximately 36 hr.

### Water As analyses

A tube well water sample was obtained from 100 of the 101 women’s primary well shortly after delivery, usually within a day, into 60-mL acid-cleaned polyethylene bottles. These were shipped to Columbia University’s Lamont Doherty Earth Observatory where they were acidified before analysis. Analytical procedures have previously been described in detail (Cheng et al. 2004). For the present study, all water samples were analyzed using an axiom single-collector high-resolution inductively-coupled plasma mass spectrometer (HR ICP-MS) (Thermo Elemental, Erlanger, Germany). The analytical detection limit of the method is 0.1 μg As/L; the standard deviation of a single measurement is conservatively estimated at 4 μg/L for concentrations up to 150 μg/L and 2% for samples > 150 μg/L (van Geen et al. 2005).

### Total As measurements

Urinary As concentrations were assayed by graphite furnace atomic absorption spectrophotometry (GFAA) using a PerkinElmer AAnalyst 600 system (PerkinElmer, Shelton, CT, USA) as described (Nixon et al. 1991). Our laboratory participates in a quality control program coordinated by P. Weber at the Quebec Toxicology Center, Quebec City, Quebec, Canada. During the course of this study, intraclass correlation coefficients between our laboratory’s values and samples calibrated at Weber’s laboratory were 0.99. The detection limit of the method is 2 μg/L. Urinary As levels were also adjusted for urinary creatinine concentrations, which were determined by a standard colorimetric method based on Jaffe’s reaction (Slot 1965), with reagents from Sigma Diagnostics (Sigma Chemical Company, St. Louis, MO, USA).

Maternal and cord venous whole blood samples were analyzed for total blood As concentration using a Perkin-Elmer Elan DRC II ICP-MS equipped with an AS 93+ autosampler as described elsewhere (Hall et al. 2006). Briefly, the As concentration of the standard solution used for instrument calibration was chosen to cover the expected range of As concentrations in the blood samples: 5, 25, and 50 μg/L. Matrix suppression was compensated for by the use of iridium as an internal standard, selected to match to the ionization potential of As. Spectral interferences for As were resolved with dynamic reaction cell (DRC) technology by introducing oxygen as a second gas. The intraclass correlation coefficients between our laboratory’s values and samples calibrated at Weber’s laboratory were 0.99. The detection limit of the method is 0.06 μg/L.

### As metabolite measurements

Urinary As and blood As metabolites were separated by high performance liquid chromatography (HPLC), with a PerkinElmer HPLC Series 200 (PerkinElmer), and then quantified by ICP-MS-DRC, using methods adapted from Reuter and Neubauer (2003). Whole blood specimens were digested using methods from Csanaky et al. (2003). Frozen samples were thawed and hemolyzed by mixing with 0.1 volume of 5.5% Triton X-100 in water. Then, 0.1 volume of 150 mM aqueous mercury chloride was added to displace trivalent As from protein thiols. After these samples were kept on ice for 1 min, they were deproteinized by mixing with one volume of 0.66 M ice cold HClO_4_ and centrifuged for 10 min at 4,000 rpm. The supernatant was mixed with mobile phase buffer and injected onto the HPLC column, connected to the ICP-MS-DRC. Calibration standards of a mixture of As metabolites were processed similar to blood samples to achieve the same pH and composition. Concentrations of calibration standards were chosen to cover the expected range of metabolite As concentrations in blood samples.

Excellent separation by HPLC coupled with very low detection limits of ICP-MS-DRC allowed us to detect MMA, DMA, As^+3^, and As^+5^, without online digestion of organic forms, with great precision in blood samples containing total As concentrations ≥ 10 μg/L. The peaks for arsenocholine (AsC) and arsenobetaine (AsB), however, could not be resolved at these low concentrations. Therefore, we report the sum value for the concentrations of AsC + AsB in blood. For quality control we used two different types of samples. We purchased blood samples with known concentrations of 23 different elements (including As) from the Institut de Sante Publique du Quebec (Quebec, Canada), for validation of ICP-MS analyses. We also created our own set of blood specimens spiked with five metabolites (AsC, As^+3^, DMA, MMA, As^+5^) in three different concentrations to cover the expected range of As in study participant samples; these were spiked at the beginning of the study, aliquoted, and frozen. We ran both sets of quality control samples at the beginning of every working day and throughout the day, after every 10 samples. Interprecision during the course of this study for AsC, As^+3^, DMA, MMA, and As^+5^ was 5.8, 7.0, 3.2, 4.5, and 9.6%, respectively.

To calculate the relative proportions of InAs, MMA, and DMA, the amounts of AsB and AsC were first subtracted from total blood As values. Because the detection limit of this method is 0.1 μg/L per individual metabolite (with a total dilution factor of 22× for blood samples), metabolite data was captured only on the 30 participants with the highest As exposure—total blood As > 10 μg/L.

### Plasma folate, B_12_, and homocysteine

We analyzed plasma concentrations of folate and total cobalamin from maternal and cord blood samples by radioimmunoassay (Quantaphase II; Bio-Rad Laboratories, Richmond, CA, USA) as described previously (Reuter and Neubauer 2003). The within- and between-day coefficients of variation for folate were 3% and 11%, respectively, and those for cobalamin were 4% and 8%, respectively. Plasma homocysteine concentrations were measured by HPLC with fluorescence detection according to the method described by Pfeiffer et al. (1999). The within- and between-day coefficients of variation for homocysteine were 5 and 8%, respectively.

### Statistical analyses

We calculated descriptive statistics for sample characteristics of mothers and their newborns. Scatterplots were used to examine the relationships between maternal and cord blood measures. We tested bivariate correlations of variables of interest based on estimated Spearman correlation coefficients, and used paired *t*-tests to detect mean difference between maternal and cord measures or between maternal blood and urine measures. We used multiple linear regression analyses to determine whether maternal folate and homocysteine influence As metabolism, after adjusting for potential confounding factors. The outcomes variables, including maternal urinary As metabolites (%DMA, %MMA, and %InAs), maternal blood As metabolites, and cord blood As metabolites (%DMA, %MMA, %As^+3^, and % As^+5^) were regressed against the main predictors variables, folate, B_12_, and homocysteine, in separate models. Folate and homocysteine data were log-transformed to normalize their distributions. Control variables for analyses of maternal urinary metabolites included maternal age, parity, gestational age indicator (< 37 weeks vs. ≥ 37 weeks), and log urinary creatinine. Control variables for maternal blood As metabolites were age and parity; for cord blood As metabolites, adjustments were made only for birth weight.

## Results

Selected characteristics of the women and their husbands and newborns are provided in Table 1. Of note, on average, both the mothers and fathers were reasonably well educated, and 89% of the mothers could read and write. There were no significant differences when these characteristics were compared between the subset for whom blood As metabolite data was obtained and the remaining 71 women (data not shown).

Mean values of water As, urinary As, and total blood As concentrations, as well as plasma folate, B_12_, and homocysteine measurements are summarized in Table 2. The mean maternal tube well water As concentration was 90.5 μg/L, with 28% of the wells exceeding the Bangladesh standard of 50 μg/L, and 38% exceeding the World Health Organization standard of 10 μg/L (Chowdhury 2004). Water As was significantly associated with both maternal and cord total blood As (*r* = 0.40, *p* < 0.0001; and *r* = 0.35, *p* = 0.0003, respectively). Water As was also significantly associated with maternal urinary As (micrograms per gram creatinine) (*r* = 0.52; *p* < 0.0001).

The mean cord total blood As values of 15.7 μg/L in the total sample (*n* = 101) and 23.13 mg/L in the subset (*n* = 30) exceeded the mean maternal total blood As of 11.9 μg/L (*n* = 101) and 20.02 mg/L (*n* = 30) by 32% (*p* < 0.0001) and 16% (*p* < 0.0001), respectively. This statistically significant finding is likely attributable to the greater concentration of hemoglobin in cord blood. The ranges for both of these parameters were extremely wide, undoubtedly reflecting, in part, the wide range of water As (Table 2). The relative proportions of As^+3^, As^+5^, MMA, and DMA were virtually identical in terms of mean values in maternal and cord blood (Table 3). In each case the mean proportions were 43% DMA, 30% MMA, 13% As^3+^, and 13% As^5+^. Compared with maternal urine (Table 3), maternal blood contained substantially higher proportions of InAs (i.e., As^+3^ + As^+5^) and MMA but less DMA. The spearman correlation coefficient between the sum of urinary As metabolites and the sum of blood As metabolites is 0.88.

For maternal and cord blood As analyses, we compared the total As concentration with the sum of As metabolite concentration values obtained by HPLC-ICP-MS. To do so, we adjusted for the HPLC percent recovery. The same approach was used to compare total urine As concentrations obtained by GFAA to those derived from the sum of urinary As metabolites via HPLC-ICP-MS. Recoveries for the HPLC-ICP-MS methods were 57% for cord blood (*n* = 30), 69% for maternal blood (*n* = 30), and 89% for maternal urine (*n* = 101). Comparison of the recovered-adjusted sum of As metabolite concentrations with the measured total As concentrations, for cord blood, maternal blood, and maternal urine, shows good agreement (Table 3) with concordance correlation coefficient of 0.87, 0.91, and 0.99, respectively.

The mean cord plasma folate and B_12_ values (52.7 nmol/L and 370.4 pmol/L, respectively) were more than twice those in maternal blood (*p* < 0.0001), whereas cord plasma homocysteine was 11% lower than maternal plasma homocysteine (*p* < 0.0001) (Table 2). Inadequate plasma folate during pregnancy, defined here as < 6.8 nmol/L (Tamura and Picciano 2006), was found in 1% of the study participants. Vitamin B_12_ deficiency, defined here as < 185 pmol/L based on nonpregnant reference values previously reported (Christenson et al. 1985), was found in 58.9% of the women in this study (Guerra-Shinohara et al. 2002; B_12_ pregnancy-specific reference ranges are not available). The prevalence of hyperhomocysteinemia was low (1.9%), based on non-pregnant cutoffs of > 10.4 μmol/L for women from the Third National Health and Nutrition Examination Survey.

Table 4 summarizes the results from multiple linear regression analyses used to determine whether maternal plasma folate and homocysteine concentrations influence As metabolism. Maternal homocysteine was a strong predictor of %DMA in maternal urine, maternal blood, and cord blood (β = −6.2, *p* < 0.02; β = −10.9, *p* < 0.04; and β = −13.7, *p* < 0.04, respectively). A positive association was also found between maternal homocysteine and urinary InAs (β = 0.84, *p* < 0.009), maternal blood As^5+^ (β = 1.3, *p* < 0.02), and cord blood As^5+^ (β = 1.3, *p* < 0.04). Maternal folate was inversely associated with maternal blood As^5+^ (β = −0.56, *p* < 0.05), and maternal B_12_ was inversely associated with cord blood As^5+^ (β = −1.2, *p* < 0.01).

We used multiple linear regression analyses to examine the predictors of maternal total blood As. We hypothesized that total blood As would decline with increasing parity, because it is known that maternal blood lead declines with parity due to the gradual “shedding” of the maternal body burden into each offspring. Because total blood As and water As had skewed distribution, these variables were log-transformed. Water As was positively associated with maternal total blood As, controlling for parity (β = 0.074, *p* < 0.0001). As hypothesized, parity was inversely associated with maternal total blood As (*r* = −0.22, *p* = 0.025), but after adjustment for water As, this association was only marginally significant (*p* = 0.094). Parity was not associated with cord total blood As before or after controlling for maternal total blood As.

**Figure 1:**
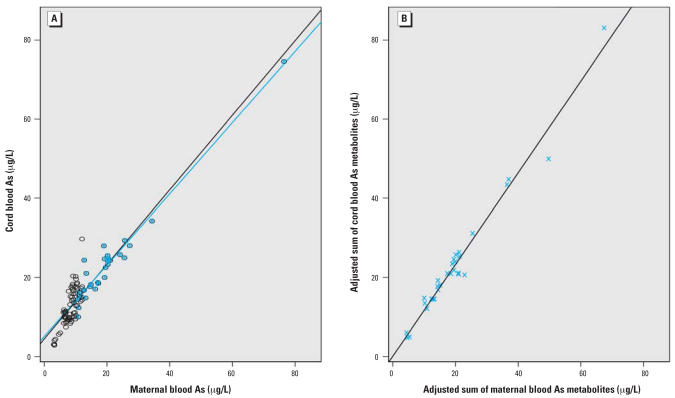
Associations between cord and maternal blood arsenic for (*A*) total blood arsenic (*n* = 30, *r* = 0.97, β = 0.90, blue line; *n* = 101, *r* = 0.93, β = 0.94, black line) and (*B*) the adjusted sum of blood arsenic metabolites (DMA + MMA + As^+3^ + As^+5^) (*n* = 30, *r* = 0.99, β = 1.16).

**Figure 2:**
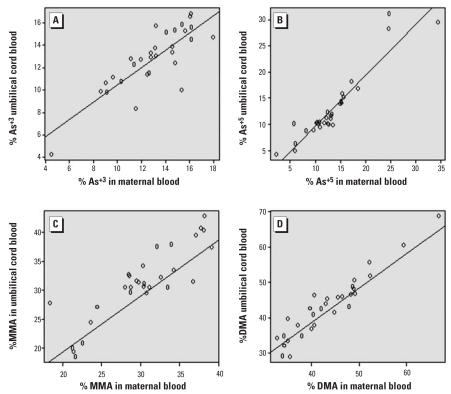
Spearman correlation coefficients (*r*) and regression coefficients (β) for pairwise comparisons of cord blood arsenic metabolites and maternal blood arsenic metabolites: As^+3^ (*r* = 0.80, β = 0.77) (*A*), As^+5^ (*r* = 0.89, β = 0.97) (*B*), MMA (*r* = 0.81, β = 0.97) (*C*), DMA (*r* = 0.94, β = 1.06) (*D*) (*n* = 30).

There was a strong association between the maternal and cord total blood As concentrations (*r* = 0.84, β = 0.94, *p* < 0.0001) (Figure 1). In the subset of 30 maternal–cord blood pairs for which blood As metabolite data were detectable, there were also strong associations for each As metabolite, as depicted in Figure 2.

## Discussion

The appearance of As in cord blood is extremely troubling because gestation is a period of high sensitivity to carcinogens (Anderson et al. 2000). Waalkes and coworkers (2004) have demonstrated that adult mice that had been briefly exposed to As during their own fetal development (during gestation days 8–18) developed tumors of the liver, ovary, adrenal gland, and lung, as well as preneoplastic lesions of the uterus and oviduct. Recently, the same group reported that when pregnant mice were exposed to As^3+^ in their drinking water, As^3+^ behaved as a complete carcinogen in male offspring, which subsequently developed liver, lung, kidney, and adrenal gland tumors. In addition, when As^3+^ exposure was combined with either *in utero* or postnatal estrogen, liver lesions were enhanced and bladder lesions also developed (Waalkes et al. 2006).

Relatively little research has examined the extent to which As exposure in pregnant women leads to exposure of the fetus to As. Two previous reports examined As concentrations in maternal–cord blood pairs. A 1991 report described findings derived from 82 births from three cities in Taiwan (Soong et al. 1991). Compared with the current study, their total blood As concentrations were low, with mean maternal and cord blood levels of only 7 and 8 μg As/L, respectively. In that low range, the authors reported a correlation between maternal and cord total blood As of 0.57 (*p* < 0.001). A second study of 11 births in northern Argentina also involved relatively low blood As concentrations and found a comparable association (Concha et al. 1998). Our present study is unique in that the study population had a very wide range of water As exposures, it employed a more sensitive and precise analytical method (i.e., ICP-MS-DRC), and, in addition to total blood As, it measured blood As metabolites, urinary As metabolites, and plasma folate, B_12_, and homocysteine concentrations. Across the range of water As exposures in this study population (0.1–661.0 μg/L), the correlation between maternal and cord total blood As was very strong (*r* = 0.84, *p* < 0.0001).

An additional concern regarding exposure to As during the prenatal period is the possible effects on birth outcomes. Hopenhayn et al. (2003) studied 844 births in two towns in Chile, one of which (Valparaiso) had water As < 1 μg/L and the other (Antofagasta) had a range of 33–53 μg/L. They reported that, after controlling for covariates, the mean birth weight in Antofagasta was 57 g lower that that in the nonexposed group, but this difference across towns did not achieve statistical significance. A much larger retrospective study of 18,259 firstborn singleton live births took place in two rural regions of Taiwan which had low and high (up to 3.59 mg/L) water As concentrations (Yang et al. 2003). After controlling for maternal age, marital status, maternal education, and sex of the infant, Yang et al. (2003) observed a significant difference in birth weight of 29 g between exposed and “nonexposed” regions (*p* = 0.002). However, the ecologic nature of this study, and its use of “extreme points of contrast” limit the ability to make causal inferences.

An additional concern about transplacental transfer of As and its metabolites during the prenatal period relates to our recent findings of a dose-dependent adverse relationship between As exposure and intelligence in young. Two separate cross-sectional studies of Bangladeshi children, one in 10-year-old children (Wasserman et al. 2004) and the second in 6-year-old children (Wasserman et al. 2007), find deficits in visual motor functioning associated with As exposure. At this point it is unclear how much of these deficits can be attributed to prenatal as opposed to postnatal exposures.

Household well water As concentrations obtained in the perinatal period proved to be predictive of both maternal and cord blood As, but these associations were considerably weaker (*r* = 0.40 and 0.35, respectively) than that with maternal urine As (*r* = 0.51). The proportion of DMA excreted in maternal urine (87%) was quite high compared with that observed in our previous cross-sectional study of men and nonpregnant women (71%) (Gamble et al. 2005b). This may be attributed partly to changes in As metabolism during pregnancy (Hopenhayn et al. 2003) or to the fact that women in the current study were supplemented with folic acid, which enhances As methylation (Gamble et al. 2006).

We observed that compared with maternal urine, maternal blood contained higher proportions of MMA and InAs and lower DMA. This finding is consistent with literature reports indicating that methylated As species have shorter half-lives of elimination than inorganic forms (Buchet et al. 1981; Vahter and Concha 2001). The literature concerning the proportions of various As metabolites in human blood is scant, but our findings are coherent with those of Pi et al. (2002), who reportedly found the proportions of InAs, MMA, and DMA to be 19.4, 49.2, and 31.4%, respectively, in adults.

Although the utility of blood As as a bio-marker of As exposure is only now being established (Hall et al. 2006), our findings do make it clear that maternal–fetal transport of As readily occurs. Importantly, the strong associations reported here between maternal and cord blood As metabolites demonstrate that the fetus is exposed to the full range of As species generated during As metabolism, although we cannot infer whether this occurs through passive diffusion of As metabolites or fetal metabolism of InAs.

Folate and homocysteine appear to be important determinants of As methylation. The importance of these parameters may be heightened during pregnancy, due to the increased demand for folate for fetal development. Animal studies have shown that insufficient folate intake decreased InAs bio-transformation and excretion, and increased tissue retention and toxicity (Spiegelstein et al. 2003; Vahter and Marafante 1987), but until recently few human studies have focused on the role of folate in As metabolism. Folate deficiency during pregnancy has been associated with various complications, including placental abruption, preeclampsia, and spontaneous abortion (George et al. 2002; Picciano 2003; Tamura and Picciano 2006), and it is well established that folic acid supplementation prevents neural tube defects and reduces the incidence of low birth weight (NRC 1998). However, in developing countries folate deficiency among unsupplemented pregnant women is still a public health concern (Tamura and Picciano 2006). Studies to delineate more fully the risks to pregnant women who are both folate deficient and exposed to InAs in drinking water should be a high priority.

The low prevalence of folate deficiency among these participants likely reflects the fact that these women received folic acid supplements during pregnancy. Our findings are also consistent with previous reports that blood folate concentrations of the newborn are significantly higher than maternal levels, presumably reflecting the *in utero* active transport process (NRC 1998). Our observation that fetal B_12_ concentrations were more than twice those of maternal levels is also in line with other reports (Molloy et al. 2002; Obeid et al. 2005; Steegers-Theunissen et al. 1997).

In summary, we have demonstrated strong relationships between maternal and cord total blood As and blood As metabolite concentrations, which indicate that newborn children in Matlab, and likely other regions of Bangladesh, are exposed to hazardous concentrations of As metabolites during the prenatal period. Given the findings reported here, we conclude that As metabolism may be impaired and its toxicity heightened in populations, such as in Bangladesh, where foods are not folic acid enriched and prenatal supplements are not always available. Interestingly, homocysteine—even at levels that are within the normal range and even after folic acid supplementation—is associated with decreased As methylation. Efforts to reduce As exposure should be a high priority, because this element has adverse effects on early childhood development, is a well-established carcinogen, and has potential synergistic effects with other co-exposures.

## Figures and Tables

**Table 1:** Characteristics of the study sample (*n* = 101).

Variable	Value	Range
Mother’s age (years)	26.0 (5.3)	16.7–39.0
Father’s age [years (*n* = 63)]	34.4 (7.0)	21.7–52.7
Mother’s education (years)	7.4 (3.6)	0–14
Father’s education (years)	7.6 (4.6)	0–16
Mother’s height (cm)	150.1 (6.0)	134–164
Parity	1.8 (0.90)	1–4
Gestational age (days)	278.4 (16.6)	218–318
Placenta weight (g)	513.6 (120.5)	300–1,030
Birth weight (g)	2752.4 (456.4)	1,160–4,000
Birth length (cm)	47.9 (2.9)	32.6–54
Head circumference [cm (*n* =100)]	31.7 (2.5)	24.2–48.2
Male births	58 (57.4)	
Female births	42 (42.6)	
Married	101 (100)	
Father’s occupation		
Unemployed	5 (5.0)	
Farmer/daily labor	24 (23.8)	
Factory worker/other paid job	22 (21.8)	
Business	21 (20.8)	
Other	29 (28.7)	
Mother able to read and write	90 (89.1)	

Values are mean ± SD or no. (%).

**Table 2:** Values for water, urine, and whole blood As and plasma folate, B_12_, and homocysteine measurements (*n* = 101).

Variable	Value	Range
Water As [μg/L (*n* = 100)]	90.5 ± 165.8	0.1–661.0
Cord blood		
As (μg/L)	15.7 ± 8.7	2.9–74.6
As [μg/L (*n* = 30)]^a^	23.1 ± 11.2	10.0–74.6
Hemoglobin (g/dL)	16.0 ± 1.6	10.8–19.4
Folate [nmol/L (*n* = 96)]	52.7 ± 16.7	24.8–106.3
Homocysteine [μmol/L (*n* = 100)]	5.7 ± 1.4	2.9–11.6
B_12_ [pmol/L (*n* = 91)]	370.4 ± 222.6	88.8–1321.1
Mother’s blood		
As (μg/L)	11.9 ± 8.6	3.1–76.5
As [μg/L (*n* = 30)]^a^	20.0 ± 12.1	10.7–76.5
Hemoglobin (g/dL)	12.7 ± 1.4	8.7–15.9
Folate [nmol/L (*n* = 99)]	20.3 ± 9.9	6.2–42.2
Homocysteine (μmol/L)	6.4 ± 1.5	3.6–10.9
B_12_ [pmol/L (*n* = 95)]	180.8 ± 71.5	84.8–458.9
Folate < 6.8 (nmol/L)	1 (1.0)	
Folate < 9 (nmol/L)	10 (10.1)	
Homocysteine > 10.4 (μmol/L)	2 (1.9)	
B_12_ < 185	56 (58.9)	
Mother’s urine		
As (μg/L)	127.6 ± 232.9	5.0–1384.0
Creatinine (mg/dL)	48.6 ± 35.3	8.6–178.9
As (μg/g creatinine)	271.7 ± 489.5	5.2–3084.4
pH (*n* = 100)	5.9 ± 0.7	5.0–7

Values are mean ± SD or no. (%).

a *n* = 30 subset is comprised of participants with total blood As > 10 μg/L.

**Table 3:** Maternal and newborn blood As metabolites and maternal urinary As metabolites for participants with total blood As > 10 μg/L (*n* = 30).

Variable	Mean ± SD	Range
Cord blood As metabolites		
%DMA	43.1 ± 8.9	28.9–68.7
%MMA	31.1 ± 6.3	18.5–42.7
%As^+3^	12.8 ± 2.7	4.3–16.8
%As^+5^	12.9 ± 6.5	4.2–31.1
Sum of As metabolites (μg/L)	24.0 ± 15.3	5.4–83.5
Total blood As (μg/L)	23.1 ± 11.2	10.0–74.6
Mother’s blood As metabolites		
%DMA	43.5 ± 8.0	32.7–66.9
%MMA	30.1 ± 5.7	18.4–39.2
%As^+3^	13.0 ± 2.9	4.5–18.0
%As^+5^	13.4 ± 6.3	2.4–34.5
Sum of As metabolites^a^ (μg/L)	20.9 ± 13.1	4.8–67.9
Total blood As (μg/L)	20.0 ± 12.1	10.9–76.6
Mother’s urine As metabolites		
%DMA	86.6 ± 5.5	74.8–97.3
%MMA	7.6 ± 4.1	1.5–17.7
%InAs	5.9 ± 2.4	1.2–11.0
Sum of urinary As metabolites^a^ (μg/g creatinine)	717.5 ± 687.1	43.4–2934.6
Total urinary As (μg/g creatinine)	726.0 ± 715.7	39.4–3084.2

a Reported sum values have been corrected for percent recovery, as described in “Methods.” Urine and blood As metabolites were measured by HPLC ICP-MS-DRC, whereas total urinary As and total blood As were measured by GFAA and ICP-MS-DRC, respectively.

**Table 4:** Estimated regression coefficients, β (SE) in models relating folate and homocysteine to maternal urinary As metabolites and maternal and cord blood metabolites.

	Main predictor variable	
Outcome variable	log (folate)	log (homocysteine)
Maternal urinary metabolites^a^ (*n* = 99)		
%DMA	−0.6 (1.4)	−6.2 (2.6)*
%MMA	1.1 (0.7)	1.4 (1.3)
%InAs	−0.1 (0.2)	0.8 (0.3)*
Maternal blood metabolites^b^,^c^ (*n* = 29)		
%DMA	3.5 (3.3)	−10.9 (5.0)*
%MMA	1.2 (2.1)	−2.1 (3.9)
%As^+3^	−0.3 (1.2)	3.5 (1.9)
%As^+5^	−0.6 (0.3)*	1.3 (0.5)*
Cord blood metabolites^b^,^d^ (*n* = 29)		
%DMA	0.1 (4.8)	−13.7 (6.2)*
%MMA	2.7 (3.7)	0.6 (5.0)
%As^+3^	0.7 (1.5)	3.8 (1.9)
%As^+5^	−0.4 (0.4)	1.3 (0.6)*

a Model was adjusted for the control variables maternal age (years), parity, indicator for gestational age (< 37 weeks vs. ≥ 37 weeks), and log (urinary creatinine).

b Unadjusted values were similar to adjusted values.

c Model was adjusted for the control variables maternal age (years) and parity.

d Model was adjusted for the control variable birth weight.

* *p* < 0.05.
